# Insulin Production and Signaling in Renal Tubules of *Drosophila* Is under Control of Tachykinin-Related Peptide and Regulates Stress Resistance

**DOI:** 10.1371/journal.pone.0019866

**Published:** 2011-05-10

**Authors:** Jeannette A. E. Söderberg, Ryan T. Birse, Dick R. Nässel

**Affiliations:** Department of Zoology, Stockholm University, Stockholm, Sweden; University of Texas MD Anderson Cancer Center, United States of America

## Abstract

The insulin-signaling pathway is evolutionarily conserved in animals and regulates growth, reproduction, metabolic homeostasis, stress resistance and life span. In *Drosophila* seven insulin-like peptides (DILP1-7) are known, some of which are produced in the brain, others in fat body or intestine. Here we show that DILP5 is expressed in principal cells of the renal tubules of *Drosophila* and affects survival at stress. Renal (Malpighian) tubules regulate water and ion homeostasis, but also play roles in immune responses and oxidative stress. We investigated the control of DILP5 signaling in the renal tubules by *Drosophila* tachykinin peptide (DTK) and its receptor DTKR during desiccative, nutritional and oxidative stress. The DILP5 levels in principal cells of the tubules are affected by stress and manipulations of DTKR expression in the same cells. Targeted knockdown of DTKR, DILP5 and the insulin receptor dInR in principal cells or mutation of *Dilp5* resulted in increased survival at either stress, whereas over-expression of these components produced the opposite phenotype. Thus, stress seems to induce hormonal release of DTK that acts on the renal tubules to regulate DILP5 signaling. Manipulations of S6 kinase and superoxide dismutase (SOD2) in principal cells also affect survival at stress, suggesting that DILP5 acts locally on tubules, possibly in oxidative stress regulation. Our findings are the first to demonstrate DILP signaling originating in the renal tubules and that this signaling is under control of stress-induced release of peptide hormone.

## Introduction

The insulin-signaling pathway is evolutionarily conserved in multicellular animals and insulin-like peptides (ILPs) regulate growth, reproduction and metabolism and play important roles in stress resistance and regulation of life span (reviewed in [1], [2], [3], [4], [5], [6]). In the fruitfly *Drosophila* genetic ablation of cells in the brain producing ILPs, or mutations in the ILP receptor (dInR) and other insulin signaling components, lead to an increase in stress tolerance and extension of life span at the expense of fertility and body size [3], [4], [6], [7], [8], [9], [10]. Also carbohydrate and lipid homeostasis is affected by these manipulations [4], [9], [10]. Seven *Drosophila* ILPs (DILP1-7) have been identified and some of these are expressed in the brain, others in fat body or intestine [5], [11], [12]. Although much has been learned about insulin signaling downstream of the insulin receptor, it is not clear how the production and release of DILPs is regulated in adult *Drosophila* in response to nutritional or stress signals [2], [4], [9]. Nutritional sensing appears to take place in adipose tissue, the fat body (see [2], [13]) and recently it was shown that there is a humoral link between the fat body and insulin-producing cells (IPCs) in the brain [14]. Thus, availability of nutrients sensed by the fat body is an important factor in regulation of DILP release. In addition recent evidence suggest that the IPCs can sense glucose levels autonomously [15].

It is likely that hormonal or neural signals also regulate production and release of DILPs by IPCs of the adult insect, as has been shown to be the case in pancreatic beta-cells in mammals (see [16], [17]). However, such hormones have not yet been identified in the fly, although recently neurons expressing, short neuropeptide F, GABA or serotonin were suggested as regulators of DILP production in IPCs of the brain [18], [19], [20]. The role of DILPs in stress responses is intriguing and we seek to investigate hormonal signaling pathways that mediate regulation of release of DILPs during stress in *Drosophila*.

For nutritional and osmotic stress one possible hormonal route is signaling from endocrine cells of the intestine. The intestine could provide further sensors to monitor metabolic status (see [21], [22]) and it has been shown that midgut endocrine cells in insects release peptide hormone at starvation [23], [24]. A few candidate peptide hormones have been identified in endocrine cells of the *Drosophila* intestine [25], [26]. We focus here on peptides encoded by the gene *Dtk* (CG14734), the five *Drosophila* tachykinin-related peptides DTKs [27], and the role of their receptors in regulation of DILPs in the fly. The reason for this focus is that we detected a novel set of cells that produce DILP5 and also express one of the two known receptors for DTKs.

We show here that the main epithelial cells of the renal tubules (Malpighian tubules), the principal cells, express both DILP5 and the DTK receptor DTKR, suggesting that these insulin-producing cells are targets of circulating DTKs. Indeed, we found that DTK signaling regulates levels of DILP5 in principal cells under nutritional stress. Since the renal tubules are not innervated, DTK can only reach them as a circulating hormone, likely to be released from the intestine (see [27]).

In *Drosophila* the renal tubules display high metabolic activity and play roles, not only in water and ion transport, but also in oxidative stress, detoxification and immune responses [28], [29], [30]. Encouraged by this and by the likely importance of insulin signaling in the physiology of the kidneys of mammals [31], [32], [33], we investigated roles of DILP5 signaling locally in the renal tubules. Interference with the expression levels of DTKR, DILP5, dInR and some further components of the insulin-signaling pathway in principal cells during metabolic and oxidative stress all lead to altered lifespan. Furthermore, knockdown of superoxide dismutase (SOD2) in principal cells leads to decreased lifespan at desiccation and oxidative stress, suggesting a possible link between insulin signaling and oxidative stress responses. We propose that insulin signaling in the tubules may be part of an autocrine regulation of renal function that in turn is controlled by hormonal DTK signaling from the intestine at metabolic and oxidative stress.

## Results

### The principal cells of the renal tubules express DILP5 and tachykinin receptors

**Figure 1:**
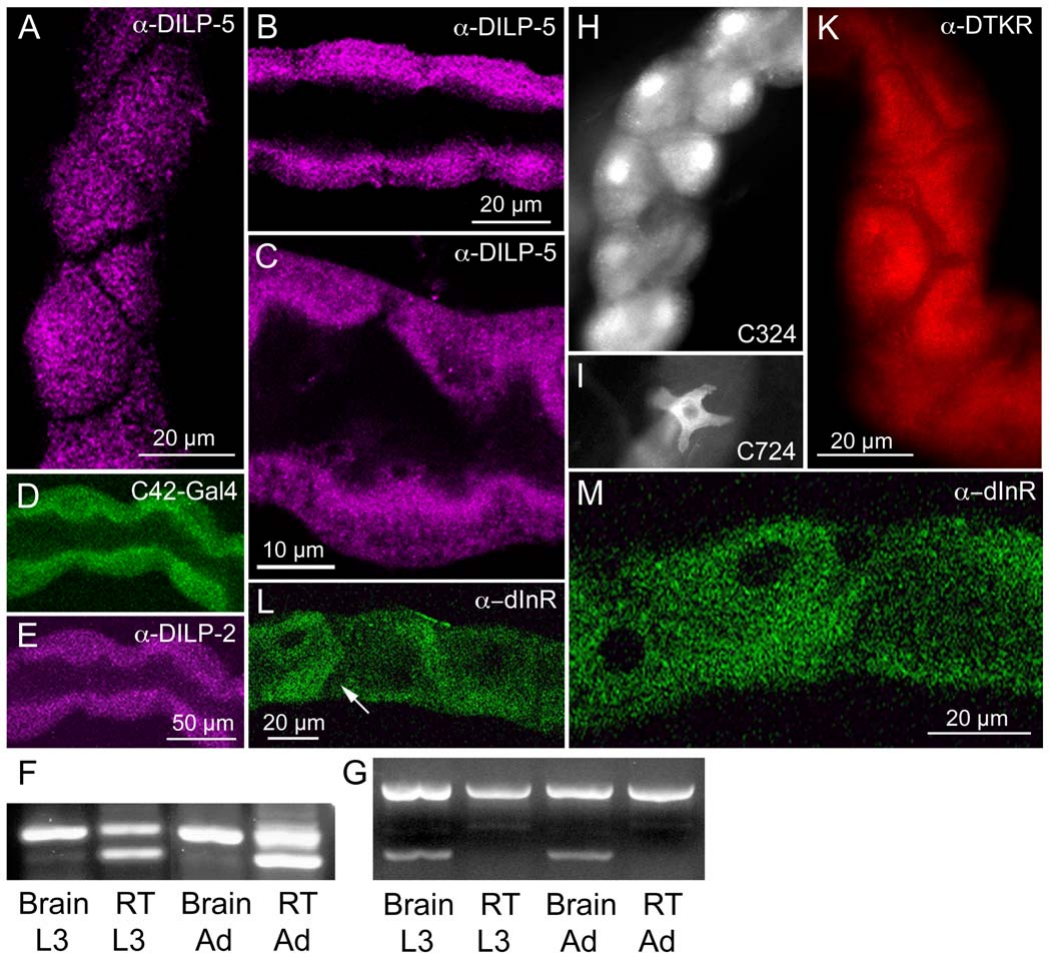
Expression of DILP5, tachykinin receptor, and insulin receptor in renal tubules. **A-C**. DILP5 immunolabeling in principal cells of adult tubules (A, B) and in tubules of third instar larva (C). A surface view is seen in D and an optical section with tubule lumen in E and F. **D, E.** Detection of DILP2 immunolabeling in the principal cells (identified by c42-Gal4-GFP in D). The antibody raised against DILP2 cross-reacts with DILP5 present in the principal cells. **F.** RT-PCR shows that *Dilp5* transcript is present both in renal tubules (RT) and brains of larvae (L3) and adults. Predicted product size is 211 bp for *Dilp5*. The bands were cut out and sequenced: the upper band in each lane represents *Dilp5* and the lower bands in RT are degenerate *Dilp-5* sequences). **G**. RT-PCR identifying *Dilp2* transcript (predicted 183 bp) in brain of larvae and adults, but not in the renal tubules. The upper band in each lane is *Rp49* transcript as loading control. Also the other *Dilp* transcripts were analyzed in the tubules; only *Dilp5* was detected (See S. Fig. 2). **H**. C324-Gal4 driven GFP expression in the principal cells. **I**. C724-Gal4 driven GFP expression is seen in the stellate cells. **K**. Immunofluorescent detection of DTKR in the principal cells in adult renal tubules. Note that the intercalating stellate cells do not express the receptor. **L, M**. Antiserum to the insulin receptor dInR labels the principal cells. Note that stellate cells are not labeled (arrow in L).

Gene microarray data has revealed enrichment of mRNA of DILP5, one of the seven known DILPs, in larval renal tubules of larval *Drosophila* (see FlyAtlas http://flyatlas.org/ [34]). Encouraged by this we developed an antiserum to the C-chain of DILP5 and show here immunolabeling of principal cells of the renal tubules in both adults and third instar larvae (Fig. 1A–C). Also an antiserum to the A-chain of DILP2 [35], that cross reacts with DILP5 due to sequence similarities, labeled these cells (Fig. 1D,E). As a control we showed that over-expression of DILP2, using the Gal4 line C324 specific for principal cells (Fig. 1H), crossed with UAS-*Dilp2,* resulted in strongly increased immunolabeling of principal cells with the DILP2 antiserum (Fig. S1A,C). This confirmed both that the antiserum recognizes ectopic DILP2 and that the principal cells can produce DILPs. We also used targeted RNA interference (RNAi) with the transgene C324-Gal4/UAS-*Dilp5*-RNAi to knock down DILP5 in principal cells and found that immunolabeling with either of the DILP2 and DILP5 antisera was strongly reduced in the tubules (Fig. S1B, C). Therefore, both the general DILP2 antiserum and the specific DILP5 antiserum recognize DILP5, and furthermore the *Dilp5*-RNAi efficiently reduces the peptide level in the principal cells. Next, we confirmed the presence of RNA encoding *Dilp5* in dissected renal tubules of larvae and adults by RT-PCR (Fig. 1F). As a comparison *Dilp2* transcript was found in the brain, but not in renal tubules (Fig. 1G). We also examined whether transcripts of the other *Dilps* are present in renal tubules and found that only *Dilp5* is present (Fig. S2)

Since our aim is to understand hormonal control of DILP release we next turned to candidate peptide receptors in renal tubules that could be involved in such regulation. Several peptide hormones are known to target renal tubules in insects to control excretory function, two diuretic hormones, leucokinin and *Capability (Capa)* -derived peptides [30]. Therefore, we first turned to peptides that may target the tubules, but have no confirmed effect on excretion. We thus searched for candidate peptide receptors on renal tubules. One of the peptide receptors, DTKR (CG7887), for the *Drosophila* tachykinins, DTKs, has previously been detected in the tubules of *Drosophila* larvae, but no function was found [36]. Here we employed an antiserum to DTKR and detected immunolabeling of principal cells also in the adult tubules (Fig. 1K). Further support for DTKR expression in renal tubules is from gene microarray data (see FlyAtlas [34]). Thus, we pursued the possible role of DTKR-mediated signaling in control of DILP release in tubules.

### DILP5 levels in principal cells are influenced by starvation and tachykinin signaling

**Figure 2:**
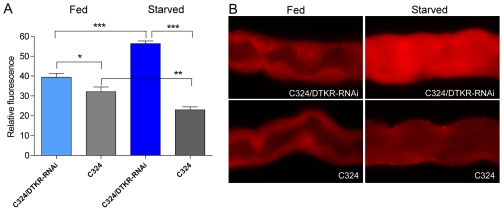
DILP levels in principal cells are altered by starvation and DTKR knockdown. **A.** The relative immunofluorescence was measured in principal cells using antiserum to the A-chain of DILP2 (known to cross react with DILP5; see Fig. S1). Each bar represents the mean relative immunofluorescence from 24 renal tubules. Knockdown of DTKR in principal cells (C324/DTKR-RNAi; light blue bar) leads to a small, but significant increase in DILP immunoreactivity compared to controls (C324; P<0.05, Student's t-test). In flies starved for 12 h the DTKR-knock down (dark blue bar) results in a higher level of DILP immunoreactivity than in controls (and fed flies) (P<0.001). Control flies (C324) starved for 12 h display weaker immunofluorescence than fed control flies (p<0.01; t-test), suggesting that starvation may induce a release of DILP5. **B–D.** Representative images of DILP immunofluorescence in renal tubules of different genotypes (DTKR knockdown and control) of fed and starved flies.

We next examined whether the production and/or release of DILP5 in tubules is dependent on DTK signaling and nutritional stress. For this we analyzed the intensity of DILP-immunofluorescence in tubules of flies that had been fed or starved for 18 h after knock down of DTKR in principal cells by C324-Gal4/UAS-*Dtkr*-RNAi. In control flies the DILP levels in principal cells decreased slightly, but significantly, after 18 h starvation (Fig. 2A). In DTKR-knockdown flies exposure to 18 h starvation resulted in significantly increased DILP fluorescence compared to fed flies of the same genotype and to the controls (Fig. 2A,B). Fed DTKR-knockdown flies also displayed DILP levels that were significantly higher than in controls (Fig. 2A). Apparently there is increased production, but possibly less release of DILP when DTKR levels are diminished, especially during starvation. We did not investigate Dilp5 RNA levels to determine whether DTK signaling affects transcription. However, our experiments show that DTKR expression levels influence DILP levels in the principal cells, especially at starvation, and we will next show that the DTK signaling influences physiological phenomena indicative of DILP signaling. In a study of the brain IPCs the levels of DILP2 immunolabeling was shown to increase at starvation, but DILP5 was unaffected [14]. However, these authors found that *Dilp5* transcript decreased when flies were fed a poor diet.

### Tachykinin signaling via DTKR affects survival in flies during metabolic stress

Since tachykinin signaling affects DILP levels we set out to test effects of interference with DTK or its receptor DTKR on possible DILP-mediated responses. Thus, we investigated the responses to stress induced by desiccation and starvation in flies carrying transgenes causing cell-specific interference with levels of DTK or DTKR. All stress experiments throughout this paper employed only male flies. In starvation experiments the flies are kept on aqueous agarose, whereas desiccated flies were deprived both food and water (thus both starved and desiccated). The specific cellular expression of the Gal4 drivers in renal tubules is shown in Fig 1H, I: the C42 and C324-Gal4, drive GFP in the principal cells and the C723-Gal4 in the smaller stellate cells (see [37], [38]).

In the first experiment we used *elav*-Gal4-driven *Dtk*-RNAi [39] to knock down the DTK peptide globally in the fly nervous system and intestine. The response of flies to desiccation was monitored as survival of transgenic flies. Flies with diminished DTK levels exhibited an extended survival time compared to controls when exposed to desiccation (p<0.001 against both controls; Log rank test; Mantel-Cox) (Fig. S3A). Desiccated control flies survive for a maximum of about 22 h with a median life span (50% survival) of about 16–18 h whereas the DTK-knockdown flies survive up to about 26 h with a median life span of about 23 h (Fig. S3A).

**Figure 3:**
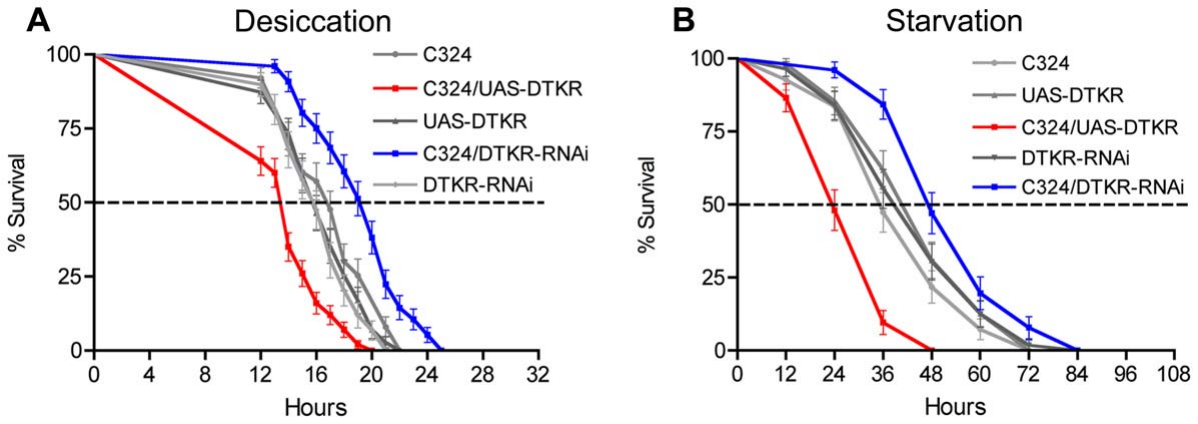
Knock down of DTKR in principal cells increases resistance to desiccation and starvation. **A**. Survival of flies with manipulated DTKR levels in principal cells (using C324-Gal4) maintained without access to food and water (desiccation). Experiments were run in three replicates (in this and subsequent figures we give n values as minimum and maximum number of flies of each genotype). A significant increase in survival (20% increase of median lifespan) was observed in flies with DTKR knockdown in principal cells (C324/DTKR-RNAi) (P<0.001 versus both parental controls, Log-rank test; n = 125–140 for the different genotypes). Conversely, over expression of DTKR in principal cells (C324/UAS-DTKR) lead to a significant decrease (14%) in survival at desiccation (P<0.001 versus both parental controls; n = 122–140). **B.** Survival of flies of the same genotypes fed 0.5% aqueous agarose (starvation). Extended survival (17%) was seen after knockdown of DTKR (C324/DTKR-RNAi) (P<0.001 versus both parental controls; n = 137–141) and a decrease (40%) in survival after over expression of DTKR in principal cells. (C324/UAS-DTKR) (P<0.001 versus both parental controls; n = 122–138). Another Gal4 driver (C42) specific for principal cells produced the same phenotypes (See Fig. S4).

We proceeded to examine the effects of over expression of the receptor, DTKR, in either of the two major cell types of the renal tubules. We used C42- or C324-Gal4 lines for principal cells and the C724-Gal4 for stellate cells to drive UAS-*dtkr*. When over expressing DTKR in the principal cells (both Gal4-lines) we detected a significant decrease in survival time of flies both during desiccation and starvation (in both cases p<0.001 for both Gal4 lines versus both controls) (Fig. 3A,B, Fig. S4), whereas over-expression in stellate cells did not alter the survival (Fig. S3A). At desiccation the over-expression of DTKR in principal cells led to a median lifespan of less than 13 h (C324) or 14 h (C42), compared to controls with 16 and 16.5 h, respectively. Starved controls display a median lifespan of about 36 h, whereas over expression of DTKR with both C42 and C324 reduced this to 24 h.

Knock down of DTKR in principal cells, with either of the two Gal4s driving *Dtkr*-RNAi, resulted in a significant increase in life span both at desiccation and starvation compared to controls (p<0.001 for both Gal4 drivers to all controls) (Fig. 3A,B). At desiccation the median life span increased from approximately 16 to 19 h. At starvation DTKR knock down in principal cells the median life span increased from 36 to 44 h with C324 and with C42 from 38 to 48 h (Fig. 2A, B; Fig. S4A, B).

Additional to DTKR there is a second DTK receptor in *Drosophila* designated NKD (CG6515; [40]). This receptor was, however, not detected in renal tubules [41]. Thus, as a control we over-expressed NKD in either the principal or the stellate cells for assays. Neither NKD genotype produced a significant effect on survival at desiccation or starvation (Fig. S3B,C).

The renal tubules are not innervated and therefore DTK can only reach them via the circulation. Based on earlier work on other insects [23], [24], we hypothesize that also in *Drosophila* DTK is released from endocrine cells of the intestine [27] at osmotic and nutritional stress and targets renal tubules. These experiments suggest that DTKR in principal cells mediates responses both to desiccation and lack of nutrition. In both cases there is an increased lifespan when diminishing DTK signaling suggesting a link to the insulin pathway.

### Increased stress resistance after DILP5 knockdown in renal tubules

**Figure 4:**
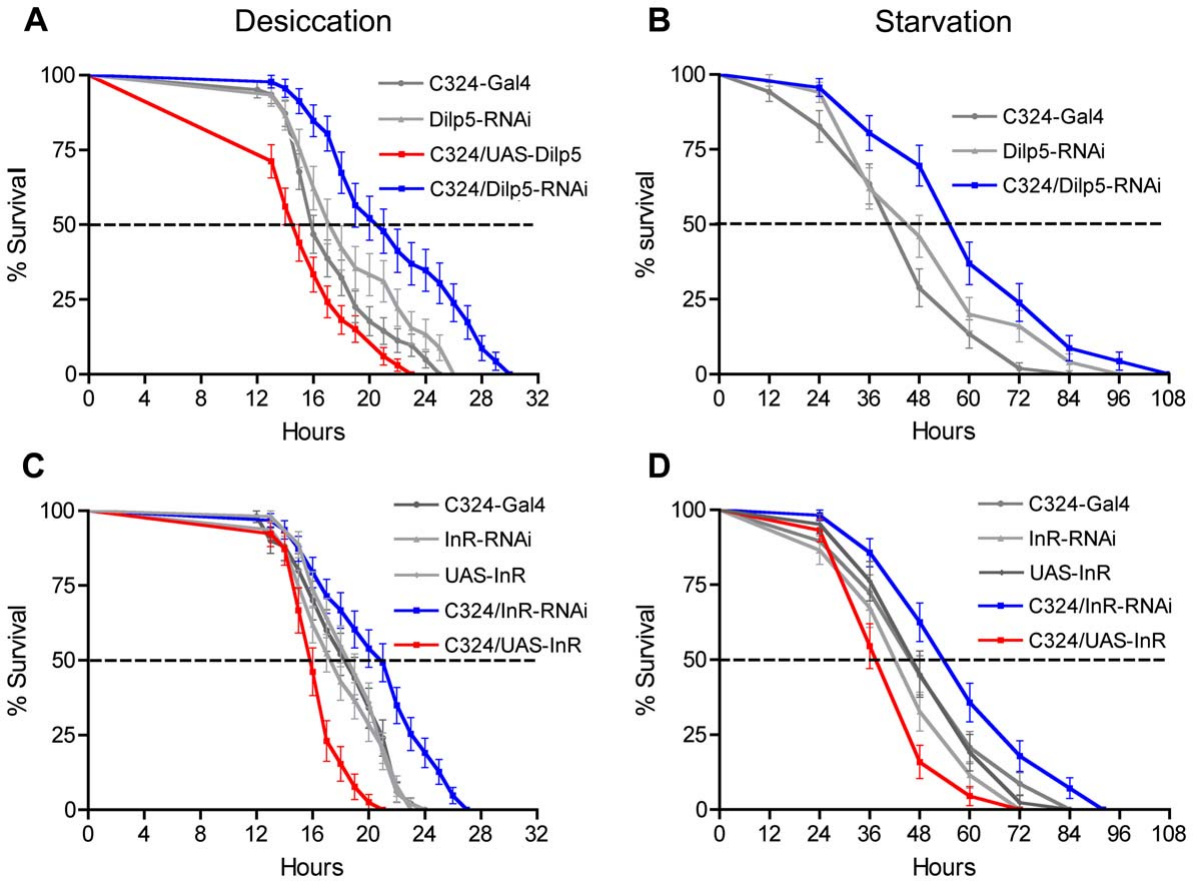
Manipulations of DILP5 and insulin receptor levels in principal cells alters survival of desiccated and starved flies. Experiments in A and B were run in triplicate, C and D in duplicate. **A**. Desiccation resistance was significantly higher after knockdown of DILP5 in principal cells (C324/*Dilp5*-RNAi); the extension of survival (median lifespan) at desiccation was 23–25%. (P<0.001 versus both parental controls; Log-rank test; n = 139–142 for the different genotypes). Over expression of DILP5 (C324/UAS-*Dilp5*) lead to abbreviated lifespan by 10–20% (P<0.01 and P<0.001 versus parental controls respectively; n = 135–148). **B**. At starvation survival is also increased after DILP-5 knockdown: C324/*Dilp5*-RNAi flies live approximately 20% longer than controls (P<0.001 versus both parental controls; n = 147–151). Another Gal4 driver (C42) specific for principal cells produced the same phenotypes (See Fig. S5). **C and D**. Knockdown of the dInR in principal cells (C324/InR-RNAi) increases survival both during desiccation and starvation. At desiccation the extension of survival for the *dInR*-RNAi flies was about 18% (P<0.001 versus both parental controls; n = 122–130) and at starvation 20% (P<0.001 versus both parental controls; n = 121–140). Over expression of the dInR in principal cells (C324/UAS-*dInR*) significantly decreases life span in both assays, by 18% (P<0.001 versus both parental controls; n = 79–101) and 17% (P<0.001 versus both parental controls; n = 70–94).

**Figure 5:**
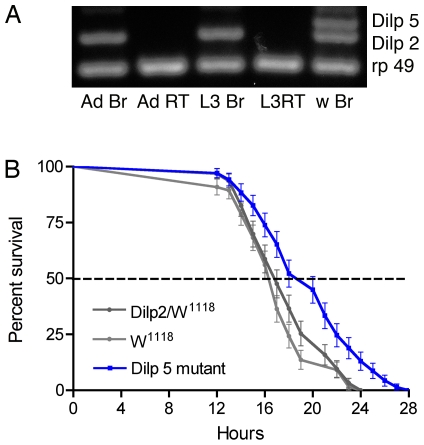
*Dilp5* mutant flies survive longer when exposed to desiccation. **A.** RT-PCR shows that *Dilp5* mutant flies lack *Dilp5* transcript both in brain (Br) and renal tubules (RT) of adults (Ad) and larvae (L3). *Dilp5* transcript is seen in the brain of wild type (w^1118^) flies (w Br). Normal transcript levels of *Dilp2* are seen in the brains of the *Dilp5* mutant compared to those of w^1118^ flies (w Br). **B.** *Dilp5* mutant flies survive longer at desiccation than controls, Dilp2-Gal4/w^1118^ and w^1118^. This experiment was run in triplicate (P<0.001 versus both parental controls; Log-rank test; n = 63–69 for the different genotypes).

Since manipulations of DTKR expression in principal cells influenced DILP levels and lifespan at stress, we proceeded to investigate the effects of direct interference with DILP5 levels on the responses to metabolic stress. Reduction of DILP5 in principal cells (using C42 and C324-Gal4 lines to drive RNAi) resulted in flies that survived significantly longer at desiccation (p<0.001 for both Gal4 drivers) and starvation than controls (p<0.001 for both Gal4 drivers) (Fig. 4A,B; Fig. S5A,B). Conversely, over-expression of DILP5 in principal cells (using C324) resulted in a significant reduction of lifespan at desiccation (P<0.001) (Fig. 4A, B). These experiments produced the same phenotypes as those obtained after interference with DTKR-mediated signaling, strengthening the proposal that DTK regulates DILP release (or at least increases DILP signaling) via DTKR. We also tested a *Dilp5* null mutant [42] for survival at desiccation. The mutant flies survived significantly longer than controls (p<0.001; Fig. 5).

### Increased stress resistance after insulin receptor knockdown in principal cells

So far we have shown that DILP5 is produced in renal tubules and is under control of DTK signaling and metabolic stress. This begs the question: what is the target of DILP5 released from these cells? According to the FlyAtlas database [34] the insulin receptor (dInR) is expressed ubiquitously in tissues, including the renal tubules. This was confirmed here with antiserum to the dInR that labels principal cells in the adult renal tubules (Fig. 1L, M).

To test whether the dInR in principal cells play a role in stress responses we drove *dInR*-RNAi [43] with the c324-Gal4 line and analyzed survival at desiccation and starvation. Flies with the dInR diminished in principal cells lived longer than controls at desiccation (p<0.001 compared to both controls) and starvation (p<0.01 to c324-Gal4 and p<0.001 to UAS-*dInR*-RNAi). Over expression of dInR in principal cells by the transgene C324-Gal4/UAS-*dInR* resulted in reduced survival both at desiccation and starvation (both p<0.001) (Fig. 4C,D).

Since global hormonal insulin signaling is known to regulate carbohydrate levels via the fat body [9], we monitored whole body trehalose levels in fed flies and after starvation for 18h after knockdown of the tachykinin receptor DTKR in principal cells (C324/*Dtkr*-RNAi). We found no difference in trehalose levels after DTKR knockdown in principal cells (Fig. S6), suggesting that, although this knockdown should influence DILP release from renal tubules, it does not impact the fat body in a detectable way. Thus, DILP5 signaling might occur locally only. We therefore suggest that DILP5 released from principal cells may act on dInRs locally in cells of the same type in an autocrine fashion. However, it cannot be excluded that DILP5 from tubules acts on additional targets, or that DILPs from other sources act on the tubules. Experiments to examine possible effects of brain-derived DILPs on principle cells, distinct from the local DILP5 signaling in tubules, might be of interest to perform in the future.

### Signaling downstream of the insulin receptor in renal tubules affects lifespan

**Figure 6:**
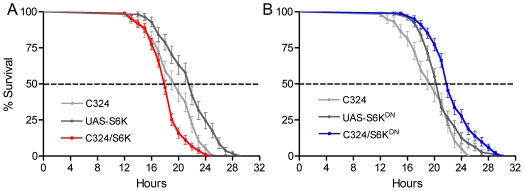
Manipulations of S6 kinase in principal cells alters survival during stress. **A**. Over expression of S6 Kinase (S6K) in principal cells by the transgene C324/UAS-S6K leads to abbreviated lifespan at desiccation by 10–20% (P<0.001 and P<0.002 to the two controls respectively; Log rank test; n = 106–118 for the different genotypes). **B**. Expression of a dominant negative form of S6K with C324 driven UAS-S6K^DN^ extends lifespan at desiccation by about 10% (P<0.001 to both controls; n = 107–115).

To monitor the effects of interference with signaling downstream of the dInR we targeted S6K (ribosomal S6 kinase) in principal cells. We over-expressed wild type S6K with the transgene C324-Gal4/UAS-*S6K*, which should phenocopy increased insulin signaling [44], [45], [46] in the principal cells. Indeed, this leads to a reduced survival at desiccation (Fig. 6A; p<0.005 and p<0.001 versus the two controls). Inactivation of S6K signaling by expression of a dominant negative construct (UAS-S6K^DN^) in principal cells produces the opposite phenotype (Fig. 6B; p<0.001). We also targeted the translational repressor 4E-BP (eukaryotic translation initiation factor 4E binding protein; Thor), known to play a role in lifespan extension at dietary restriction, starvation and oxidative stress in *Drosophila* [47], [48]. Expression of a mutant form of 4E-BP with increased activity (4E-BP^LL^) in principal cells resulted in increased lifespan at desiccation (Fig. S7A; p<0.01), whereas over expression of the wild type form did not significantly affect lifespan (Fig. S7B).

**Figure 7:**
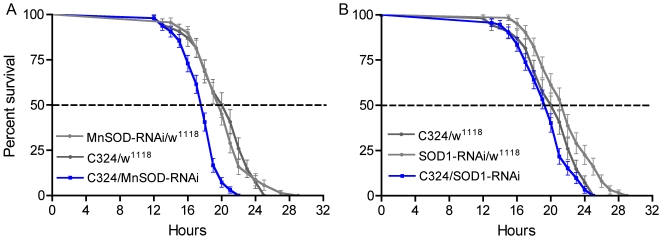
Knockdown of superoxide dismutase in renal tubules diminishes survival during stress. **A**. Knock-down of mitochondrial manganese superoxide dismutase (MnSOD; SOD2) in principal cells (C324/MnSOD-RNAi) and exposure of flies to desiccation reduced lifespan significantly (P<0.001 to both parental controls; Log rank test; n = 64–96 for the different genotypes). **B.** Knockdown of cytoplasmic CuZnSOD (SOD1) in principal cells (C324/SOD1-RNAi) did not produce a strong phenotype at desiccation (P<0.01 compared to SOD1-RNAi/w^1118^ and P = 0.1 to C324/w^1118^; n = 64–92 for the different genotypes).

Oxidative stress is a major factor in the process of aging [49], [50]. Renal tubules are known to be involved in responses to oxidative stress [29], [51]. In fact, knock down of a mitochondrial inner membrane ATP/ADP exchanger, ANT, in principal cells of renal tubules is sufficient to reduce survival of the fly at oxidative stress [29]. Mitochondrial respiration is a major source of reactive oxygen species and one defense against oxidative stress is superoxide dismutase 2 (SOD2; MnSOD) located in mitochondria [52]. Transcript of *Sod2* (CG8905) is enriched in adult renal tubules (FlyAtlas) and therefore tested the effects of knocking down *Sod2* in principal cells on survival at desiccation. Flies with the transgenes C324/*sod2*-RNAi displayed significantly reduced lifespan at desiccation (Fig. 7A; P<0.001). We also crossed C324 flies to UAS-*Sod1*-RNAi to test whether the cytoplasmic CuZnSOD (SOD1, CG11793) plays a role in the principal cells. Flies of this cross did not differ from controls in their response to desiccation (Fig. 7B; P = 0.1), in congruence with FlyAtlas data showing no enrichment of *Sod1* transcript in renal tubules. At present we have no evidence that DILP signaling affects SOD2 activity, although knockdown of both in principal cells affect survival at desiccation.

**Figure 8:**
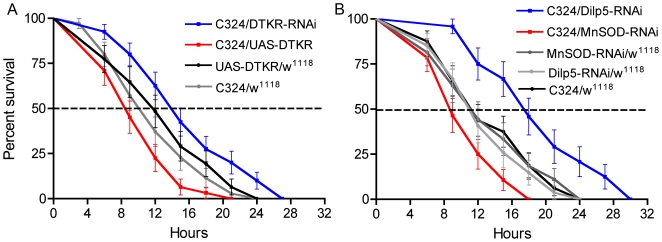
Responses to paraquat-induced oxidative stress in transgene flies. Flies were kept on standard food with 20 mM paraquat in tests of lifespan during oxidative stress. Each experiment was run in two replicates. **A.** Over-expression (C324/UAS-DTKR) and knock down (C324/DTKR-RNAi) of the DTK receptor DTKR in principal cells affects survival during oxidative stress. A significant increase in survival is seen with diminished receptor (P<0.004 and P<0.01 compared to parental controls; Log rank test; n = 50–60 for the different genotypes) and the opposite phenotype was obtained after over-expression of DTKR (P<0.009 and P<0.001 compared to the two controls; Log rank test; n = 50–60 for the different genotypes). **B.** Knockdown of *Dilp5* (C324/DILP5-RNAi) and *Sod2* (C324/MnSOD-RNAi) in principal cells also significantly affects survival at oxidative stress. Knockdown of *Dilp5* leads to a drastic increase in median and total lifespan (P<0.001 and P<0.0005 compared to parental controls; n = 50–60 for the different genotypes) whereas *Sod2*-RNAi decreases lifespan (P<0.01 and P<0.02 compared to parental controls; n = 50–60 for the different genotypes).

The finding that SOD2 activity in renal tubules plays a critical role in the survival of flies during desiccation urged us to investigate the role of DTKR and DILP signaling during oxidative stress. Thus, we fed flies standard food containing 20 mM of paraquat to induce oxidative stress. Knockdown of DTKR in the principal cells increased lifespan in flies fed paraquat (P<0.01 and P<0.004 compared to controls), whereas over expression of the receptor decreased it (P<0.01 and P<0.001) (Fig. 8A). We also fed paraquat to flies with *Dilp5* and *Sod2* knockdown in principal cells. The *Dilp5* RNAi drastically increased the survival, whereas *Sod2* RNAi abbreviated lifespan at oxidative stress (*Dilp5* RNAi, P<0.001 compared to controls; *Sod2* RNAi, P<0.01 and P<0.02 to the controls) (Fig. 8B). These findings support that renal tubules play an important role in defense against oxidative stress and that DTK and DILP signaling may be involved in this defense.

It can be noted that we have no evidence for DTK signaling to renal tubules directly affecting fluid secretion. We did, however, monitor the ability of transgenic flies to retain water at desiccation. Over expression of DTKR in principal cells significantly increased water loss (P<0.001 to controls), whereas knock down of the receptor produced the opposite effect (P<0.001 and P<0.01 to controls) (Fig. S8). These finding suggest that DTK receptor signaling also affects the diuretic activity of the tubules.

### DILP5 signaling in larval renal tubules

**Figure 9:**
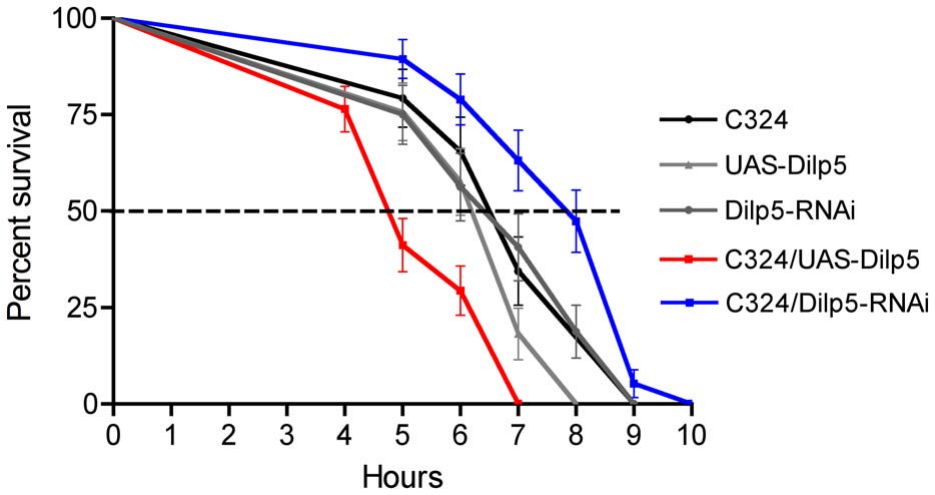
Manipulations of DILP5 expression in larval tubules also affect lifespan during stress. Over expression or knockdown of DILP5 in principal cells of feeding third instar larvae also affects survival at metabolic stress. Larvae were kept without food on a wet filter paper. Experiments run in triplicate. Knockdown by C324/Dilp5-RNAi increased median lifespan by almost 25% (P<0.01 to both controls; Log rank test; n = 30 for the different genotypes) and over expression by C324/UAS-Dilp5 decreased lifespan by the same amount (P<0.001 to both controls; n = 30 for the different genotypes).

Finally, since *Dilp5* RNA and peptide are enriched also in larval renal tubules (Fig. 1F, L) (see also FlyAtlas; [34]), we investigated whether local insulin signaling contributes to lifespan regulation in larvae. We used the C324 driver to diminish or over-express *Dilp5* in the renal tubules of feeding third instar larvae (Fig. 9). Control larvae that were kept on a wet filter paper and no access to food displayed a median lifespan of about 6.5 h. With increased DILP5 they displayed a reduction by 1.5 h (p<0.001) whereas with diminished DILP5 lifespan increased by the same time (p<0.01). This suggests that also in the feeding larvae DILP5 signaling in the renal tubules plays a role in metabolic stress responses.

## Discussion

We have identified the renal tubules as a novel site of insulin production and signaling in *Drosophila*. The principal cells of these tubules produce DILP5 and express the ubiquitous DILP receptor, dInR. From our findings we suggest that DILP5 may signal locally within the epithelium of the renal tubules. This local DILP signaling appears to be under hormonal regulation during desiccative, nutritional and oxidative stress by means of the peptide DTK acting on the receptor, DTKR, localized on the principal cells. Our findings that diminished DTKR, DILP5 and dInR extend life span suggest an involvement of this signaling pathway in tubules in desiccation, nutritional and oxidative stress responses in adult *Drosophila*. Finally, manipulations of dS6K, 4E-BP and SOD (SOD2) in principal cells altered life span of flies at stress supporting that insulin signaling acts within the tubules, probably in regulation of oxidative stress responses. Interestingly, the signaling within the renal tubules affects the survival of the whole organism as shown also for mitochondrial function in tubules at oxidative stress [29].

The roles of DILPs in stress resistance and regulation of life span are well established in *Drosophila* [3], [7], [10], but hormonal mechanisms for regulation of production and release of DILPs in IPCs of adult flies have not been reported. Thus our demonstration of DTKs acting on IPCs in the renal tubules is a first identification of a hormonal factor regulating DILP release in adult insects. Interestingly, there is evidence for actions of tachykinins on IPCs also in mammals: the tachykinin substance P has been shown to increase insulin secretion from the pancreas of rat and pig and this effect is reversed in the diabetic rat [53], [54].

Since the renal tubules are not innervated, peptide receptors in this tissue can only be activated by hormonal messengers. One source of hormonal DTKs in *Drosophila* is a population of endocrine cells in the intestine (midgut) located close to the attachment of the renal tubules [27]. In locust and cockroach similar cells have been identified and it was shown that at starvation tachykinin-related peptide was released into the circulation [24].

Renal tubules in insects have been primarily investigated with respect to their function in water and ion transport and several peptide hormones have been implicated in the control of diuresis [30]. Our findings here suggest that peptide hormones that target the renal tubules may play roles other than in direct regulation of diuresis. The *Drosophila* renal tubules express an impressive array of genes and combined with experimental analysis it is suggestive that this tissue partakes in detoxification processes, oxidative stress, dietary osmotic stress and immune responses [28], [29], [34], [55], [56], [57], [58].

How does DTK signaling to the renal tubules produce a response that affects sensitivity to desiccation and starvation? The DTK signal may be a general metabolic stress signal that reaches the renal tubules. In our experiments this stress signaling is amplified with the over-expression of DTKR in principal cells and diminished by its knockdown leading to changes in lifespan. The role of DTK may be to regulate factors in principal cells involved in local metabolism, oxidative stress resistance or immune responses at the cost of decreased life span when in over-drive. One such a factor may be DILP5. Both in *Drosophila* and *C. elegans* immune response genes are expressed in the intestine (including renal tubules in the fly) and recent work has shown that these genes are under control of insulin signaling [59], [60], [61]. In *Drosophila* the DILP signaling pathway is involved in infection-induced wasting (loss of energy stores) where reduced signaling leads to reduction in pathology [61].

Also oxidative stress resistance is linked to insulin signaling in *Drosophila* (see [3], [48], [62]). Superoxide dismutases (SOD) are key enzymes protecting proteins from reactive oxygen species and are thought to be regulated by insulin signaling: SOD activity is elevated in *chico* (dInR substrate) mutants of *Drosophila* [7], [62] and Daf-2 mutants of *C. elegans* [63]. Also in yeast insulin-signaling mutations affect lifespan via SOD [64]. We found here that knockdown of *Sod2* (encoding MnSOD), but not *Sod1*, in renal tubules decreased lifespan at desiccation and oxidative stress in *Drosophila*. Thus, it is possible that DILP signaling in tubules target mitochondrial SOD2 and affects resistance to oxidative stress. Interestingly, diminishing oxidative stress resistance via *Sod2* locally in the principal cells of *Drosophila* renal tubules is sufficient to shorten the lifespan of the fly during stress. This is similar to findings in a study of genetical impairment of a mitochondrial inner membrane ATP/ADP exchanger in the same cells [29],

In conclusion, this study presents evidence for DTK controlled insulin signaling in the renal tubules of *Drosophila* being important for survival at metabolic and oxidative stress. Our findings may suggest an autocrine regulatory loop within the tubules with a role in renal function. Local signaling within *Drosophila* renal tubules has previously been demonstrated with endogenously produced tyramine [65] and nitric oxide [58], that regulate chloride permeability and innate immune responses, respectively. It is possible that the insulin signaling in the renal tubules is part of the epithelial immune system or oxidative stress defense via SOD, but we cannot exclude that the dInRs on principal cells regulate DILP5 production or release and that additional DILP5 targets are located outside the renal tubules.

## Materials and Methods

### Fly stocks

All flies were grown on a diet of yeast-cornstarch-agar medium, under 12∶12 light:dark conditions, and at a temperature of 25°C. For immunocytochemistry we used *Drosophila melanogaster* of the strains Oregon R and w^1118^, or for special purposes transgenic flies described below. A number of *Drosophila* lines were used for experiments (the complete genotypes are given in the original references listed). For global knockout of Dilp5 we used a *Dilp5* mutant, w*; *dilp5^1^,* [42] kindly provided by L. Partridge and S. Grönke (London, UK). This mutant was generated by ends-out homologous recombination and was shown to be a specific DILP5 protein null allele [42]. The enhancer trap Gal4 lines specific for principal cells (C324-Gal4 and C42-Gal4) and the stellate cells (C724-Gal4) [37], [38] were donated by J. Dow and S. Davis (Glasgow, UK). An *Elav*-Gal4 (C155) was obtained from Bloomington *Drosophila* Stock Center (at Indiana University, Bloomington, IN). The UAS-*Dilp5* and UAS-*Dilp2* [5], [66] were provided by P. Shen (Athens, GA) and UAS-*Dilp5*-RNAi (CG33273; code 49520) was obtained from the Vienna *Drosophila* RNAi Center (VDRC, Vienna, Austria). The UAS-*dInR*-RNAi (18402-R1 and 18402-R2) lines [43], [67] were provided by J. R. Martin (Gif sur Yvette, France). An RNAi construct for DTK precursor (*Dtk*) knockdown, UAS-*Dtk*-RNAi37D, stably crossed to *Elav-Gal4* to generate the *Elav;;*UAS-*Dtk*-RNAi37D strain [39], was provided by Å. Winther (Stockholm, Sweden) and the UAS-*Dtkr*-RNAi and UAS-*Dtkr* constructs were described previously [68]. The lines UAS-*S6K* and UAS-*S6K^KQ^* (dominant negative S6K) [69] and UAS*-4E-BP^wt^* and UAS*-4E-BP^LL^* [47] were obtained from the Bloomington *Drosophila* Stock Center. *4E-BP^LL^* is a mutated 4E-BP with increased binding. We used two RNAi lines for knockdown of superoxide dismutase (SOD). One was for *Sod1* (CuZnSOD; CG11793, [70]) and another for *Sod2* (MnSOD; CG8905, [52]). These flies (Codes 108307 and 42162 respectively) were from VDRC. A UAS-*Nkd* strain was produced for this investigation as outlined in Text S1. Parent strains were used as controls throughout this study.

### Reverse transcription–polymerase chain reaction analysis (RT–PCR)

Renal tubules were dissected from anesthetized flies in insect saline and immediately processed using the Trizol protocol from Invitrogen, to extract mRNA. cDNA was synthesized by RT-PCR using the One Step RT-PCR Kit from Qiagen. The primers for *Dilp5* were: 5′ AGTTCTCCTGTTCCTGATCC 3′ and 3′CAGTGAGTTCATGTGGTGAG 5′ and for Dilp2 they were 5′ GTA TGGTGTGCGAGGAGTAT 3′ and 3′ TGAGTACACCCCCAAGATAG 5′ [18]. For control we employed *rp49*, using the primers 5′ GTATCGACAACAGAGTCGGTCGC 3′ and 5′ TTGGTGAGCGGACCGACAGCTGC 3′. Further primers used in Fig. S2 are given in Text S1.

### Immunocytochemistry and microscopy

Rabbit antiserum to a portion of the C-chain (YEDHLADLDSSESHH) of *Drosophila* DILP-5, conjugated N-terminally to thyroglobulin, was generated by Pineda Antibody Service (Berlin, Germany). The antiserum was applied to tissue fixed in 4% paraformaldehyde in sodium phosphate buffer. Antiserum was diluted (1∶2,500) in 0.01 M PBS with 0.5% BSA and 0.25% Triton X-100 and incubation was for 48h at 4°C. Secondary antiserum was goat anti-rabbit tagged with cyanamide (Cy3; Jackson ImmunoResearch, West Grove, PA, USA) at 1∶1,500. This protocol was also followed for the antisera listed below. A rabbit antiserum to the A-chain of DILP2 [35] was a gift from M. R. Brown (Athens, GA). This was applied at a dilution of 1∶1000. For detection of the tachykinin receptor DTKR we used a rabbit antiserum to a portion (AAs 488–506) of the C-terminus of the receptor [36], applied at 1∶2,000. A rabbit antiserum to the *Drosophila* insulin receptor (dInR), raised against a fusion protein of a dInR sequence [71], was used at 1∶1,000 (provided by J. Mattila and O. Puig; Helsinki, Finland). Microscopic analysis was performed on a Zeiss Axioplan 2 microscope coupled to a CCD camera (Zeiss AxioCam HRc, Jena, Germany) or a Zeiss LSM 510 confocal microscope.

### Quantification of immunofluorescence

Immunocytochemistry with DILP-2 antiserum was performed on renal tubules from starved and fed flies for quantification of immunofluorescence. Images were obtained with a Zeiss Axioplan 2 microscope with Axiovison software (fixed exposure time 728 ms), and immunofluorescence was quantified in a set of regions of interest (14300 pixels), using Image J 1.40 from NIH, Bethesda, Maryland, USA (http://rsb.info.nih.gov/ij/). The data were analyzed in Prism GraphPad 6.0, with Student's t-test.

### Assays of life span during starvation, desiccation and oxidative stress

Male flies, aged 4–8 days, were anesthetized using CO_2_ and placed individually in 2 ml glass vials kept in an incubator with 12∶12 LD light conditions at 25°C and controlled humidity. The starvation experiments were performed following the protocol of Lee and Park [72]. The tubes were supplied with 500 µl of 0.5% aqueous agarose. For the desiccation experiments flies were kept in tubes with neither food nor water. For starvation experiments the vials were checked every 12 h and for desiccation tests after 12 h and then every 1 h. To induce oxidative stress we fed flies standard food containing 20 mM paraquat (methyl viologen, Sigma, St Louis) as described in [10]. Flies were kept in vials with 0.5 ml of this food mixture and survival was checked every 3 h. In all the above experiments survival curves and statistics (Log rank test; Mantel-Cox) were made using Prism GraphPad 5.0.

## Supporting Information

Figure S1 **Relative DILP immunofluorescence in principal cells after interference with DILP expression. A**. Over expression of DILP-2 (C324/UAS-DILP2) drastically increases the DILP-2 immunolabeling in principal cells (representative images are shown in A and B). **B.** Knockdown of DILP-5 by C324/UAS-*Dilp5*-RNAi strongly reduced DILP-2 immunolabeling. The DILP-2 antiserum was raised against the A-chain which is more conserved between DILPs and thus likely to recognize also DILP-5. The loss of fluorescence suggests that the antiserum indeed recognizes DILP-5, the only likely DILP in these cells. This experiment also indicates that the *Dilp5*-RNAi causes a decrease in peptide in principal cells. **C**. Relative immunofluorescence levels in principal cells comparing over expression of DILP-2 and knock down of DILP-5 with C324-Gal4 control. Over expressing DILP-2 in the principal cells significantly increased the immunofluorescence labeling whereas knocking down DILP-5 significantly decreases the immunosignal (*** P<0.001). Based on measurements of 6 tubules of each genotype.
(TIF)

Figure S2 **RT-PCR of extracts from renal tubules identifies only** ***Dilp5*** **transcript. A.** Extracts of renal tubules were assayed with primers to *Dilp2-7* with *rp49* as a loading control. Only *Dilp5* was detected. Experiment was run in duplicate. **B.** As a control the same primers were applied to extract of whole heads. All the Dilps were detected. The Dilp2 and 5 samples were extracted separately and thus appear weaker (as seen by rp49 expression).
(TIF)

Figure S3 **Survival of transgenic flies exposed to desiccation** **or starvation.** Experiments in this figure were run in at least duplicate, with a minimum of 40 flies of each genotype. **A**. Flies with global knockdown of DTK peptide (*Tk*-KO) by means of Elav-Gal4/*Dtk*-RNAi display an increased life span at desiccation (P<0.001 compared to *elav*-Gal4 and other genotypes;, Log-rank test). The median life span (50% survival) increased by about 43%. Several other transgenes did not affect the response to stress. Ectopic expression of the other DTK receptor NKD in stellate cells, using the cross C724/UAS-*NKD*, or in principal cells (C324-Gal4/UAS-NKD) did not alter survival compared to controls, (P>0.05 to parental controls), neither did the ectopic expression of DTKR in stellate cells (C724/UAS-*DTKR*) (P>0.05 to parental controls). **B and C.** Ectopic expression of NKD in principal cells has no effect on response to desiccation or starvation. Flies with NKD expression in principal cells by C324-Gal4 (C324/UAS-*NKD*) or C42-Gal4 (C42/UAS-*NKD*) do not display any alterations of life span at desiccation (**B**; P>0.05 to parental controls), nor during starvation (**C**; P>0.05 to parental controls).
(TIF)

Figure S4 **DTKR interference in principal cells using a different Gal4 driver (C42) also affects response to starvation and desiccation. A**. Flies were subjected to desiccation and their survival was measured. Flies expressing *DTKR*-RNAi in principal cells by means of the C42-Gal4 driver (C42/DTKR-RNAi) increased their median life span by about 3 hours (p<0.001 versus both parental controls; Log rank test; n = 124–130 for the different genotypes; triplicate). Flies over expressing DTKR (C42/UAS-DTKR) displayed an approximately 3 h shorter lifespan than the controls (p<0.001 versus both parental controls; n = 126–140). **B**. At starvation the effects of DTKR knockdown and over expression on life span are the same as at desiccation. Flies over expressing DTKR in principal cells display reduced survival and flies expressing *DTKR*-RNAi live longer compared to controls (p<0.001 for each transgene compared to both parental controls; Log rank test; n = 125–140; triplicate).
(TIF)

Figure S5 **Survival of flies after interference with DILP-5 levels in principal cells using a different Gal4 driver (C42).** Survival rates after desiccation (**A)** and starvation (**B**). Knock down of DILP-5 in the principal with the C42-Gal4 driver leads to a longer life span at desiccation (P<0.001 versus both parental controls, Log rank test, n = 125–135 for the different genotypes) and at starvation (P<0.001 versus both parental controls, n = 124–132).
(TIF)

Figure S6 **Trehalose levels in different genotypes exposed to desiccation or starvation.** Whole body trehalose levels measured from transgenic flies before and after 18 h of starvation. 40 flies for each genotype were analyzed in two replicates. At 0 h, flies were fed and watered normally and thereafter subjected to starvation for 18 h. We tested over expression and knockdown of DTKR (DTKR-RNAi) in principal cells (C42-Gal4) and over expression of DILP-2 (UAS-DILP-2) in the same cells. Controls are shown in grey bars, experimental ones in colored bars. All genotypes displayed a drastic drop (50% or more) in trehalose levels after 18 h starvation. However, no significant difference in change of trehalose levels could be detected between the different genotypes, suggesting that the DTKR signaling in the renal tubules does not primarily influence whole body trehalose levels.
(TIF)

Figure S7 **Altered survival of flies exposed to desiccation after manipulation of 4E-BP in principal cells.** Flies with expression of an active form of 4E-BP (Thor) by the transgene C324-Gal4/UAS-4E-BP^LL^ increased life span significantly compared to the two controls (P<0.01 versus both parental controls; Log rank test; n = 99–138 for the different genotypes; experiment run in duplicate). However, flies with wild type 4E-BP over expressed in principal cells with the transgene C324-Gal4/UAS-4E-BP^WT^ did not display a significant change in life span at desiccation.
(TIF)

Figure S8 **Water loss during desiccation in flies with altered DTKR expression.** The graph shows percentage water loss in whole flies after 12 h of desiccation in transgene flies in comparison to controls (UAS-DTKR, C42, DTKR-RNAi). A minimum of 40 flies was used for each genotype for this assay (run in triplicate). Flies over expressing DTKR in principal cells (C42/UAS-DTKR) displayed a greater loss of water over 12 hours (red bar) than controls (grey; P<0.001), whereas flies with knock down of DTKR in principal cells (C42/DTKR-RNAi) showed a reduced loss of water (blue bar) compared to its parental controls (*** P<0.001 and **P<0.01)**.**
(TIF)

Text S1 Supporting information text.
(DOC)
