# Supplementary figures and images for: Insulin Production and Signaling in Renal Tubules of Drosophila Is under Control of Tachykinin-Related Peptide and Regulates Stress Resistance
Source: PLoS One. 2011 May 10;6(5):e19866. doi: 10.1371/journal.pone.0019866 (PMC3091884; doi:10.1371/journal.pone.0019866)

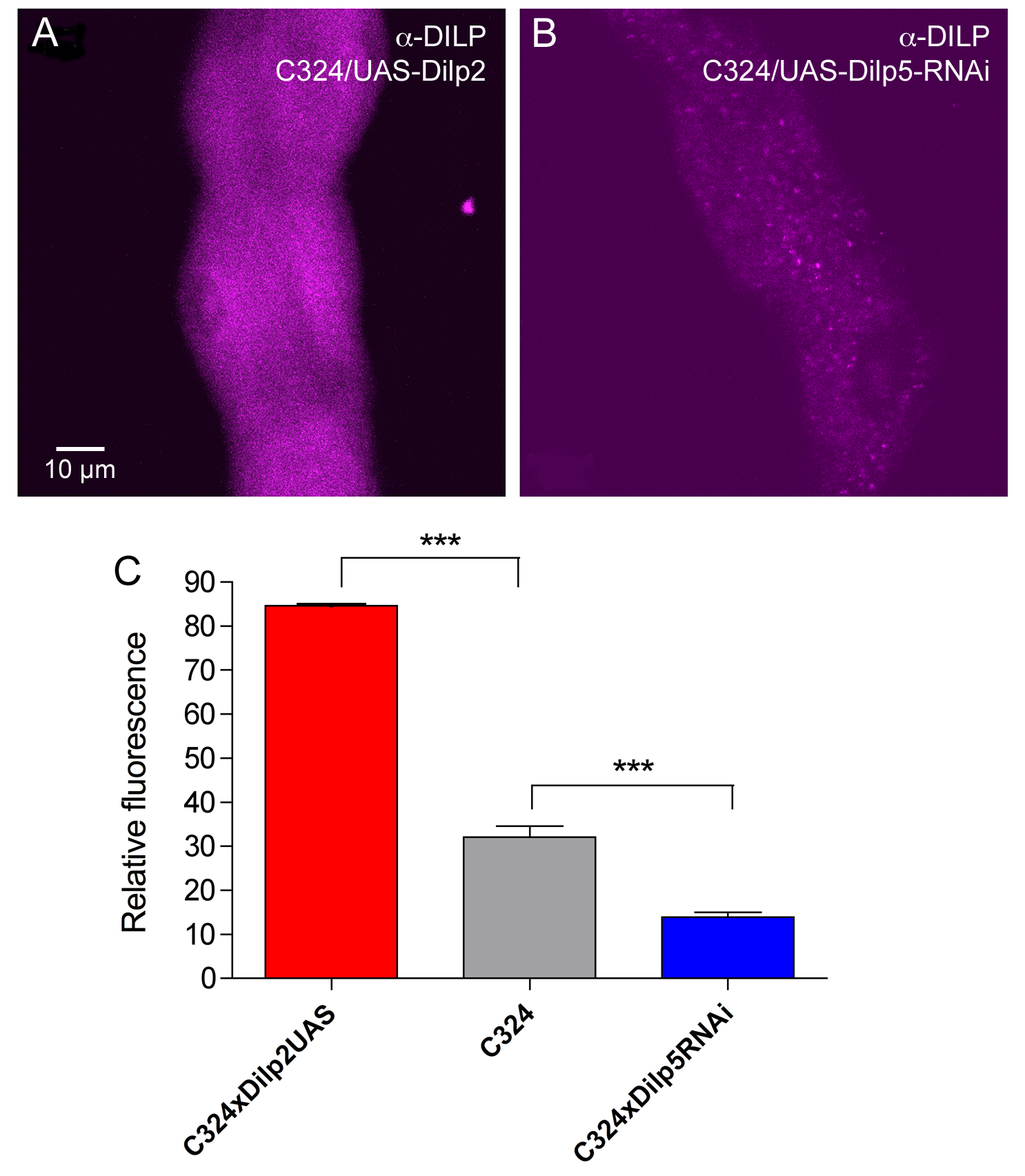

Supplement: Figure S1 — Relative DILP immunofluorescence in principal cells after interference with DILP expression. A. Over expression of DILP-2 (C324/UAS-DILP2) drastically increases the DILP-2 immunolabeling in principal cells (representative images are shown in A and B). B. Knockdown of DILP-5 by C324/UAS-Dilp5-RNAi strongly reduced DILP-2 immunolabeling. The DILP-2 antiserum was raised against the A-chain which is more conserved between DILPs and thus likely to recognize also DILP-5. The loss of fluorescence suggests that the antiserum indeed recognizes DILP-5, the only likely DILP in these cells. This experiment also indicates that the Dilp5-RNAi causes a decrease in peptide in principal cells. C. Relative immunofluorescence levels in principal cells comparing over expression of DILP-2 and knock down of DILP-5 with C324-Gal4 control. Over expressing DILP-2 in the principal cells significantly increased the immunofluorescence labeling whereas knocking down DILP-5 significantly decreases the immunosignal (*** P<0.001). Based on measurements of 6 tubules of each genotype. (TIF) [file pone.0019866.s001.tif]

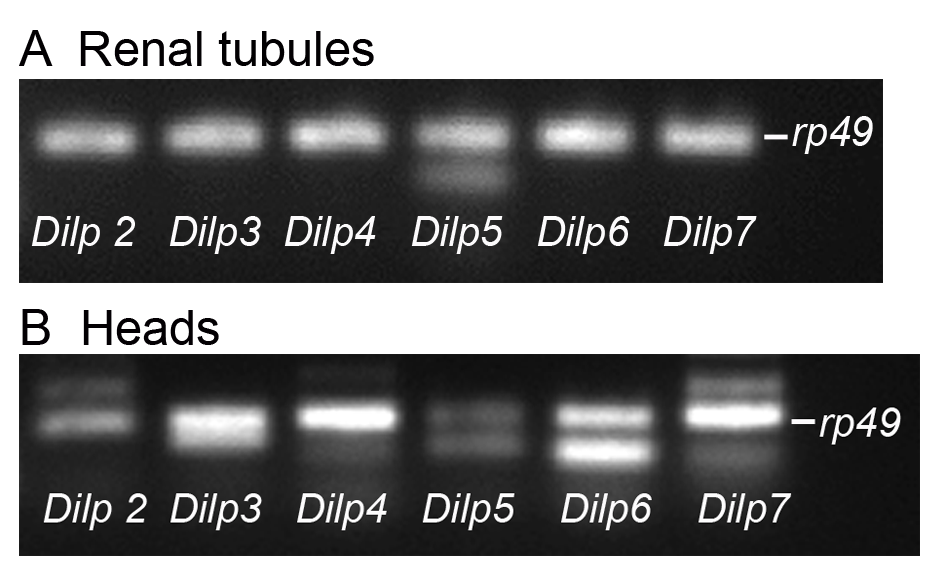

Supplement: Figure S2 — RT-PCR of extracts from renal tubules identifies only Dilp5 transcript. A. Extracts of renal tubules were assayed with primers to Dilp2-7 with rp49 as a loading control. Only Dilp5 was detected. Experiment was run in duplicate. B. As a control the same primers were applied to extract of whole heads. All the Dilps were detected. The Dilp2 and 5 samples were extracted separately and thus appear weaker (as seen by rp49 expression). (TIF) [file pone.0019866.s002.tif]

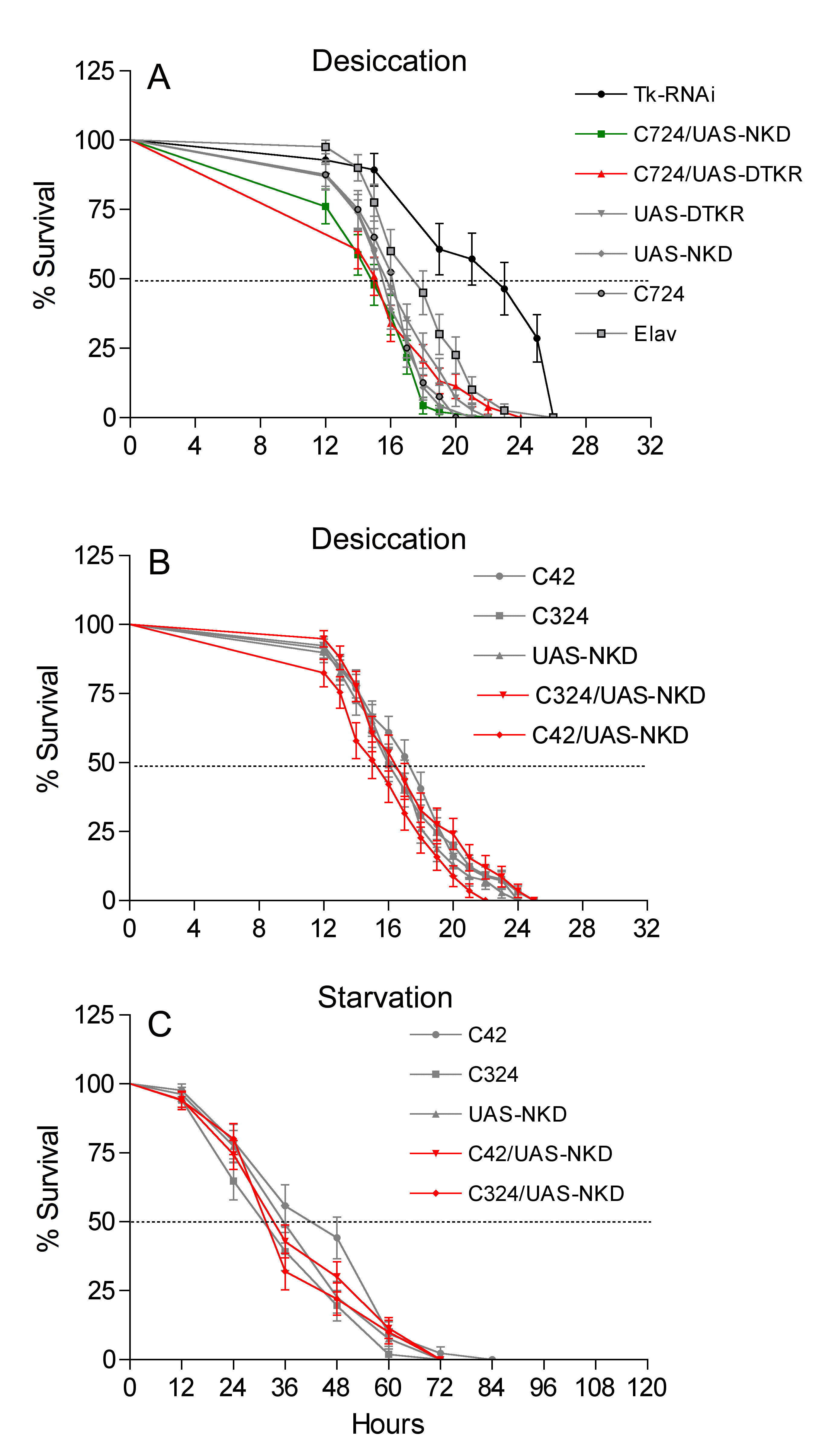

Supplement: Figure S3 — Survival of transgenic flies exposed to desiccation or starvation. Experiments in this figure were run in at least duplicate, with a minimum of 40 flies of each genotype. A. Flies with global knockdown of DTK peptide (Tk-KO) by means of Elav-Gal4/Dtk-RNAi display an increased life span at desiccation (P<0.001 compared to elav-Gal4 and other genotypes;, Log-rank test). The median life span (50% survival) increased by about 43%. Several other transgenes did not affect the response to stress. Ectopic expression of the other DTK receptor NKD in stellate cells, using the cross C724/UAS-NKD, or in principal cells (C324-Gal4/UAS-NKD) did not alter survival compared to controls, (P>0.05 to parental controls), neither did the ectopic expression of DTKR in stellate cells (C724/UAS-DTKR) (P>0.05 to parental controls). B and C. Ectopic expression of NKD in principal cells has no effect on response to desiccation or starvation. Flies with NKD expression in principal cells by C324-Gal4 (C324/UAS-NKD) or C42-Gal4 (C42/UAS-NKD) do not display any alterations of life span at desiccation (B; P>0.05 to parental controls), nor during starvation (C; P>0.05 to parental controls). (TIF) [file pone.0019866.s003.tif]

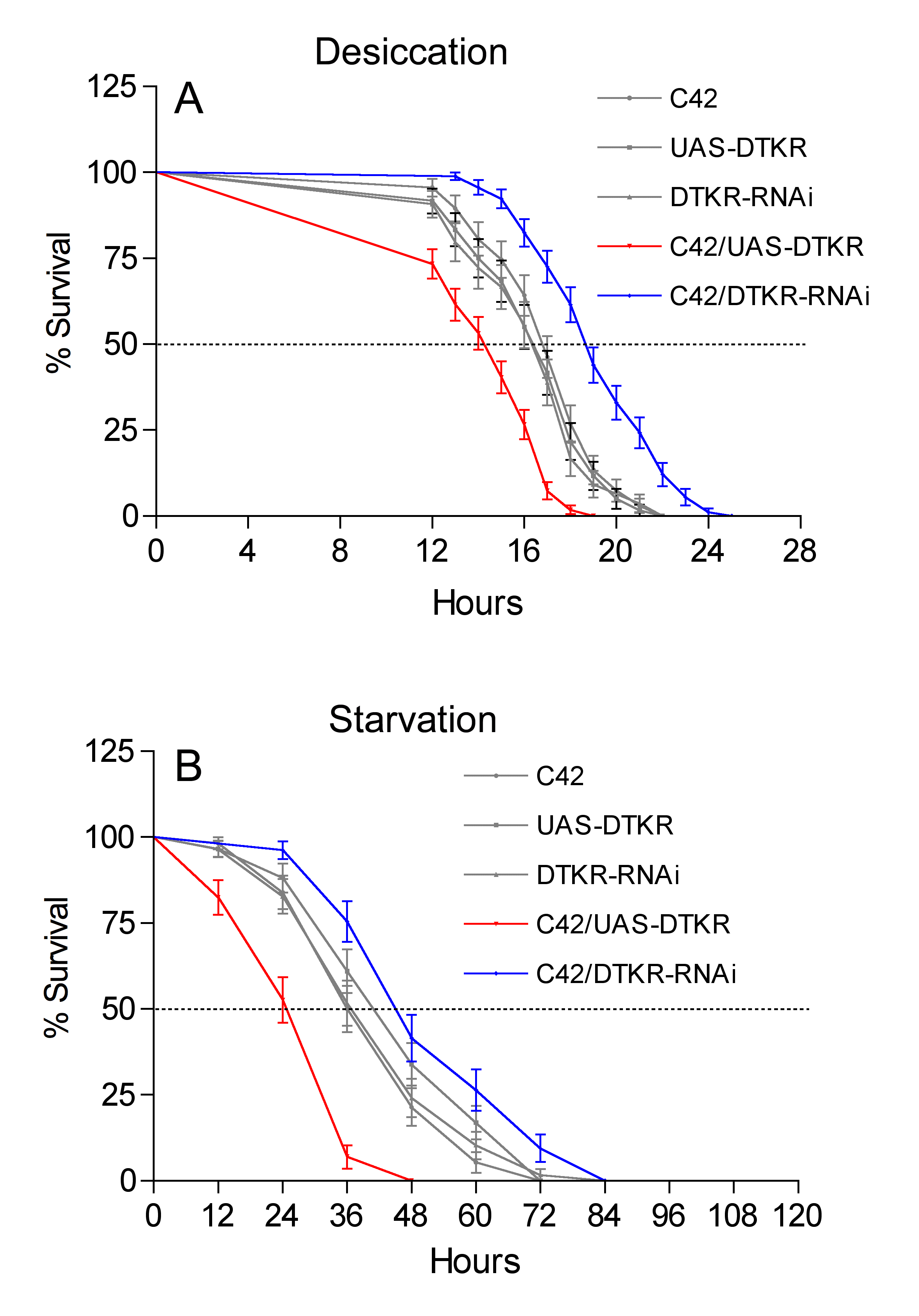

Supplement: Figure S4 — DTKR interference in principal cells using a different Gal4 driver (C42) also affects response to starvation and desiccation. A. Flies were subjected to desiccation and their survival was measured. Flies expressing DTKR-RNAi in principal cells by means of the C42-Gal4 driver (C42/DTKR-RNAi) increased their median life span by about 3 hours (p<0.001 versus both parental controls; Log rank test; n = 124–130 for the different genotypes; triplicate). Flies over expressing DTKR (C42/UAS-DTKR) displayed an approximately 3 h shorter lifespan than the controls (p<0.001 versus both parental controls; n = 126–140). B. At starvation the effects of DTKR knockdown and over expression on life span are the same as at desiccation. Flies over expressing DTKR in principal cells display reduced survival and flies expressing DTKR-RNAi live longer compared to controls (p<0.001 for each transgene compared to both parental controls; Log rank test; n = 125–140; triplicate). (TIF) [file pone.0019866.s004.tif]

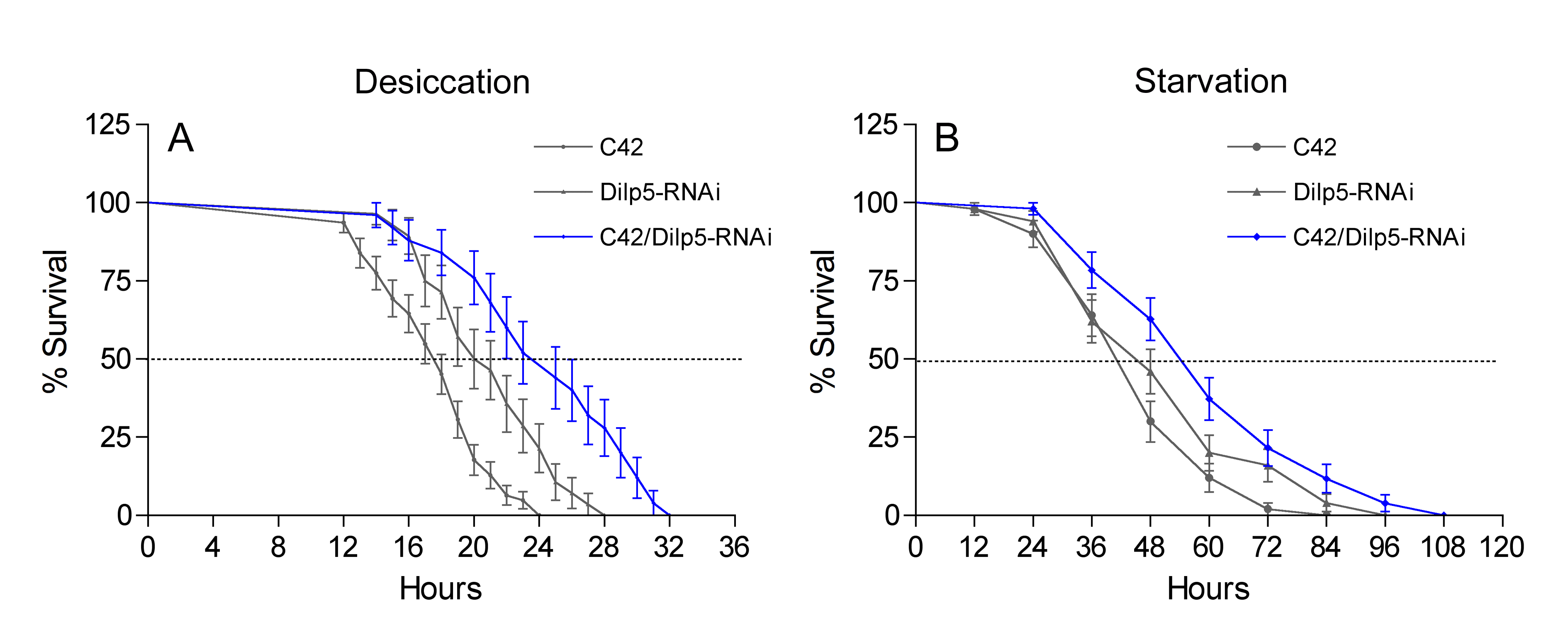

Supplement: Figure S5 — Survival of flies after interference with DILP-5 levels in principal cells using a different Gal4 driver (C42). Survival rates after desiccation (A) and starvation (B). Knock down of DILP-5 in the principal with the C42-Gal4 driver leads to a longer life span at desiccation (P<0.001 versus both parental controls, Log rank test, n = 125–135 for the different genotypes) and at starvation (P<0.001 versus both parental controls, n = 124–132). (TIF) [file pone.0019866.s005.tif]

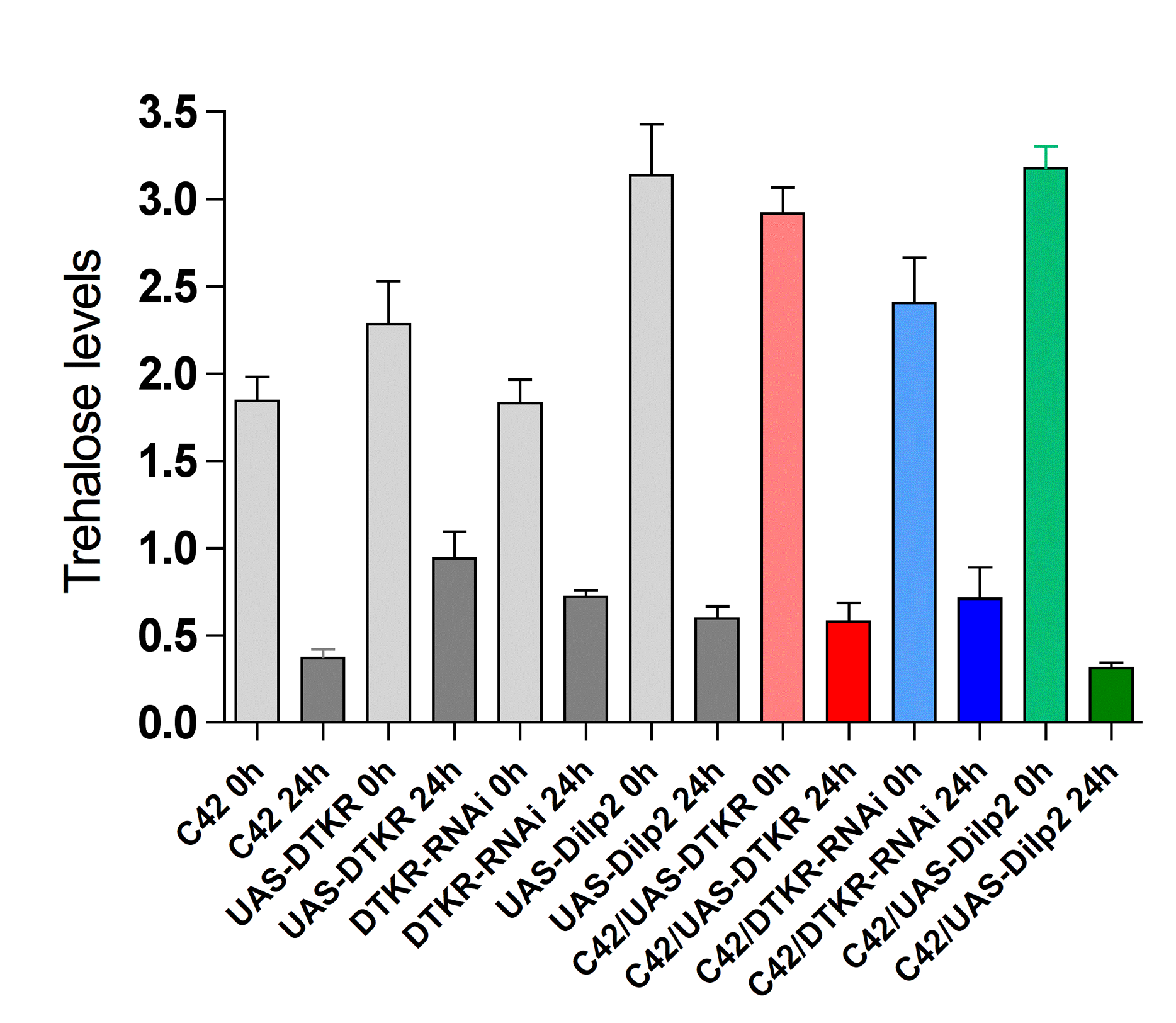

Supplement: Figure S6 — Trehalose levels in different genotypes exposed to desiccation or starvation. Whole body trehalose levels measured from transgenic flies before and after 18 h of starvation. 40 flies for each genotype were analyzed in two replicates. At 0 h, flies were fed and watered normally and thereafter subjected to starvation for 18 h. We tested over expression and knockdown of DTKR (DTKR-RNAi) in principal cells (C42-Gal4) and over expression of DILP-2 (UAS-DILP-2) in the same cells. Controls are shown in grey bars, experimental ones in colored bars. All genotypes displayed a drastic drop (50% or more) in trehalose levels after 18 h starvation. However, no significant difference in change of trehalose levels could be detected between the different genotypes, suggesting that the DTKR signaling in the renal tubules does not primarily influence whole body trehalose levels. (TIF) [file pone.0019866.s006.tif]

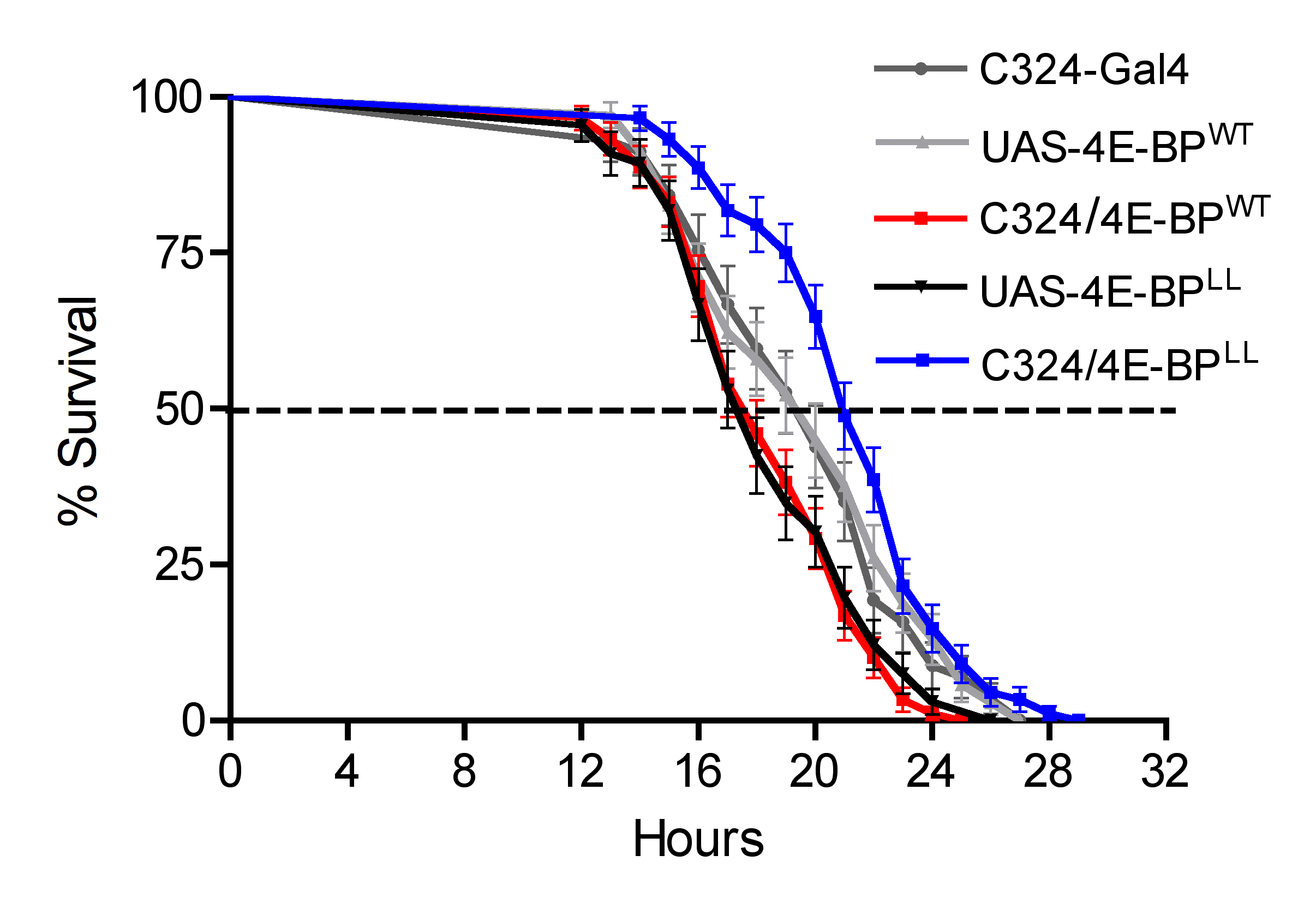

Supplement: Figure S7 — Altered survival of flies exposed to desiccation after manipulation of 4E-BP in principal cells. Flies with expression of an active form of 4E-BP (Thor) by the transgene C324-Gal4/UAS-4E-BPLL increased life span significantly compared to the two controls (P<0.01 versus both parental controls; Log rank test; n = 99–138 for the different genotypes; experiment run in duplicate). However, flies with wild type 4E-BP over expressed in principal cells with the transgene C324-Gal4/UAS-4E-BPWT did not display a significant change in life span at desiccation. (TIF) [file pone.0019866.s007.tif]
