# Supplementary material for: Insulin Production and Signaling in Renal Tubules of Drosophila Is under Control of Tachykinin-Related Peptide and Regulates Stress Resistance
Source: PLoS One. 2011 May 10;6(5):e19866. doi: 10.1371/journal.pone.0019866 (PMC3091884; doi:10.1371/journal.pone.0019866)
Supplement: Text S1 — Supporting information text. (DOC) [file pone.0019866.s009.doc]

**Supporting INFORMATION Text (Text S1)**

METHODS

**Generation of transgenic flies**

A UAS-*Nkd-GFP* fusion construct, to express the tachykinin receptor NKD, was designed according to Birse et al. [1] (see also [2]). A *Bglll/Notl* fragment containing the GPCR sequence (CG6515) and the GFP fusion was excised from the pEGFP1 vector. This was subcloned into a pUAST vector (donated by Paul Taghert, University of Washington, MO). The primer sequences for NKD were:

5’ GCGCAAGCTTGCCGCCACCATGTCGGAGAT

3’ GGTACCGGCATCTGCTTGGGACTGAG.

The constructs was injected into embryos of *w^1118^* flies following standard procedures for P-element germ-line transformation [3]. From these injections several strains were established. However, only UAS*-nkd-GFP(3b)* (referred to as UAS*-*NKD) strain was used for the experiments presented here. The strain is homozygous viable and was found to display strong green fluorescence when crossed to the Gal4 drivers. The receptor-GFP fusion is functional and could be activated by the DTK ligands in cell expression systems [1].

**Primers for RT-PCR**

Primers used for RT-PCR of renal tubules and head in S. Fig. 2 are listed below. Two sets of primers were used for rp49 due to transcript size overlapping those of *Dilps* in some cases. Primers were from [4,5]

Dilp 2 Forward

GTATGGTGTGCGAGGAGTAT

Dilp 2 Reverse

TGAGTACACCCCCAAGATAG

Dilp3 Forward
AAGCTCTGTGTGTATGGCTT

Dilp3 Reverse
AGCACAATATCTCAGCACCT

Dilp 4 Forward
GCGGAGCAGTCGTCTAAGGA

Dilp 4 Reverse
TCATCCGGCTGCTGTAGCTT

Dilp 5 Forward

AGTTCTCCTGTTCCTGATCC

Dilp 5 Reverse

CAGTGAGTTCATGTGGTGAG

Dilp 6 Forward
CGATGTATTTCCCAACAGTTTCG

Dilp 6 Reverse
AAATCGGTTACGTTCTGCAAGTC

Dilp 7 Forward
CAAAAAGAGGACGGGCAATG

Dilp 7 Reverse
GCCATCAGGTTCCGTGGTT

rp49 Forward
AGGGTATCGACAACAGAGTG

rp49 Reverse
CACCAGGAACTTCTTGAATC

rp49 Forward (second primer)

GTATCGACAACAGAGTCGGTCGC

rp49 Reverse (second primer)

TTGGTGAGCGGACCGACAGCTGC

**Measurements of trehalose levels**

Whole body trehalose was measured according to Isabel et al. [6] with a few minor alterations. In brief, male flies (4-8 days old) were pooled in groups of 5 flies per tube. Flies were weighed (wet weight), then incubated for one hour in 500 µl of 70% EtOH. Each tube of flies was sonicated (Sonics and Materials Inc. Danbury CT. USA) for 20 seconds. 1 ml of 70% EtOH was added, tubes were vortexed briefly and incubated at room temperature for one hour. A trehalose standard was made with a 2 fold dilution series starting at 200µg/ml. The samples were centrifuged for 5 min at x 13,200 rpm and 1 ml of the samples and 500 µl of the standards were placed in 2 ml Eppendorf tubes. Samples were dried in a vacuum centrifuge (Savant Speed Vac; Speed Vac Plus Sc110A). To each tube 200µl of 2% NaOH was added and vortexed. After mixing, samples well, 1.5 ml of fresh Anthrone reagent (Sigma; Cat. #A 1631) was added and vortexed until the sample had a homogenous yellow color. Samples were then placed in a water bath set at 90°C for 10 minutes. After this incubation period the samples were removed and 100µl of each sample was placed in a 96 well ELISA plate. Each sample was measured in triplicate on an ELISA plate reader (Labsystems, Multiscan Plus).

###### Measurement of water content and loss

Male flies were exposed to 12 hours desiccation to determine water loss after DTKR knockdown or over expression (and in parental strains). Two groups of flies of each genotype were weighed: (1) normally fed flies (0 hour desiccation) and (2) after 12 hours desiccation with no food and no water (12 hours desiccation). To obtain water content groups of 5 male flies were weighed (Mettler MT5) after anesthetizing them on ice (living wet weight) and were subsequently dried at 60°C for 24 hours. Dry flies were weighed after reaching room temperature (dry weight). Water content was calculated by subtracting the dry weight from the wet weight. Water loss over 12 h was calculated for each genotype by subtracting the water content at 12h from that at 0h. Since dead dry weight was necessary to obtain we had to use separate flies for 0h and 12h. Experiments were run in triplicate with at least 40 flies of each genotype and replicate.

**References**

1. Birse RT, Johnson EC, Taghert PH, Nässel DR (2006) Widely distributed *Drosophila* G-protein-coupled receptor (CG7887) is activated by endogenous tachykinin-related peptides. J Neurobiol 66: 33-46.

2. Ignell R, Root CM, Birse RT, Wang JW, Nässel DR, et al. (2009) Presynaptic peptidergic modulation of olfactory receptor neurons in *Drosophila*. Proc Natl Acad Sci U S A 106: 13070-13075.

3. Robertson HM, Preston CR, Phillis RW, Johnson-Schlitz DM, Benz WK, et al. (1988) A stable genomic source of P element transposase in *Drosophila melanogaster*. Genetics 118: 461-470.

4. Grönke S, Clarke DF, Broughton S, Andrews TD, Partridge L (2010) Molecular evolution and functional characterization of Drosophila insulin-like peptides. PLoS Genet 6: e1000857.

5. Lee KS, Kwon OY, Lee JH, Kwon K, Min KJ, et al. (2008) *Drosophila* short neuropeptide F signalling regulates growth by ERK-mediated insulin signalling. Nat Cell Biol 10: 468-475.

6. Isabel G, Martin JR, Chidami S, Veenstra JA, Rosay P (2005) AKH-producing neuroendocrine cell ablation decreases trehalose and induces behavioral changes in *Drosophila*. Am J Physiol Regul Integr Comp Physiol 288: R531-538.
